# Modelled cost-effectiveness analysis of the Support and Treatment After Replacement (STAR) care pathway for chronic pain after total knee replacement compared with usual care

**DOI:** 10.1186/s12962-024-00532-5

**Published:** 2024-04-11

**Authors:** Sophie Cole, Sian Noble, Rachael Gooberman-Hill, Rafael Pinedo-Villanueva

**Affiliations:** 1 Nuffield Department of Orthopaedics, Rheumatology and Musculoskeletal Sciences, Nuffield Orthopaedic Centre, University of Oxford, Windmill Road, OX3 7LD Oxford, UK; 2 Bristol Medical School, University of Bristol, 1-5 Whiteladies Road, BS8 1NU Bristol, UK; 3 Bristol Medical School, University of Bristol, Royal Fort House, BS8 1UH Bristol, UK; 4 National Institute for Health Research Bristol Biomedical Research Centre, University Hospitals Bristol and Weston NHS Foundation Trust and University of Bristol, Bristol, UK; 5 National Institute for Health Research Oxford Biomedical Research Centre, University of Oxford, Oxford, UK

**Keywords:** Cost-effectiveness analysis, Post-surgical pain, Total knee arthroplasty

## Abstract

**Background:**

The aim of the study was to estimate the long-term cost-effectiveness of the Support and Treatment After Replacement (STAR) care pathway for chronic pain after total knee replacement compared with usual postoperative care.

**Methods:**

Study design: A decision-analytic (cohort Markov) model was used for the simulation with time dependent annual transition probabilities and a time horizon of five years.

Setting: Patients treated by National Health Service (NHS) hospitals in England and Wales.

Study population: Adults classified as having chronic pain three months after undergoing a total knee replacement.

Intervention: The STAR care pathway following a total knee replacement.

Comparator: Usual postoperative care following a total knee replacement.

Perspective: The study was undertaken from the perspective of the NHS.

Outcome measures: Quality-adjusted life years and healthcare costs.

Discounting: A rate of 3.5% for both costs and health utility.

**Results:**

Model results indicate that the STAR intervention would dominate current practice by providing a gain in quality-adjusted life years (QALYs) of 0.086 and a reduction of £375 (per person) in costs over the first five years. The incremental net monetary benefit of the STAR intervention was estimated at £2,086 (at a threshold of £20,000 per QALY). Probabilistic sensitivity analysis suggests the STAR intervention is likely to be cost-effective with a probability of 0.62.

The results remain robust to changes in model assumptions on comparator utility and the timing of the start of the intervention. If hospital admission costs are assumed not to be reduced by the STAR intervention, it would no longer be cost saving, but it would likely be cost-effective based on probabilistic sensitivity analysis (0.59).

**Conclusion:**

Evidence from the economic model suggests that the STAR care pathway is likely to be cost-effective and potentially dominant from an NHS perspective.

**Trial registration:**

The STAR trial is registered with ISRCTN, ISRCTN92545361.

**Supplementary Information:**

The online version contains supplementary material available at 10.1186/s12962-024-00532-5.

## Background

Many people who undergo a total knee replacement (TKR) experience a significant improvement in health-related quality of life and reduction in pain post-operation. However, for some, pain is present and persistent long after the operation with some estimates placing the proportion of people undergoing surgery who then report chronic pain at 20% [1–3]. Post-surgical chronic pain is classified as pain which persists for three or more months after the operation [4, 5].

The Support and Treatment After Replacement (STAR) care pathway [6] is an intervention designed to reduce pain severity experienced by individuals after a total knee replacement. This care pathway was refined over time through multiple stages of work including (but not limited to) a systematic review [7], a survey of current practice, and the contribution of expert opinion (patient and public involvement, clinicians, and academic researchers) through consensus questionnaires, discussion meetings, and dry runs of the assessment clinic to test the intervention. The STAR care pathway includes, at three months post-operation: a one-hour clinical assessment for pain, neuropathic pain, and depression (through patient-reported outcome measures, PROMs), as well as a detailed inspection of the knee. Following the clinical assessment, individuals are referred to appropriate services that could target and help to alleviate pain, and that are already available as part of the National Health Service (NHS) [6]. Referrals were personalised to the individual’s needs. Supporting individuals with chronic pain post-operation could be an effective way to utilise more fully services already provided by the NHS and improve their quality of life. TKR is a common operation offered by the NHS with over a 100,000 surgeries performed each year [8]. As a result, any improvement in the care provided to individuals who undergo a TKR, even if small, could have a large positive impact in healthcare service efficiency and patients’ quality of life.

The within-trial economic evaluation found the STAR intervention to be cost-effective in the short-term (12 months’ follow-up) [8, 9]. The aim of our study was to further examine the cost-effectiveness of the STAR care pathway when compared to usual postoperative care over a 5-year period and using data obtained directly from the original trial combined with other real-world data sources.

## Methods

### Intervention, study population and settings

The STAR care pathway comprises a clinical assessment by a healthcare professional (with specialist orthopaedic training). Based on this assessment, the STAR care pathway provides personalised care according to individual needs, as patients are referred to appropriate existing NHS services such a physiotherapist, orthopaedic surgeon, or a general practitioner as required [8]. Patients may also receive up to six follow-up calls with a trained extended scope practitioner during the 12-month duration of the intervention. The STAR care pathway was designed through multiple phases of work including refinement of the intervention, testing the delivery of the intervention, and an evaluation of its execution. This work was completed by a wide range of individuals, incorporating viewpoints from public and patient representatives, healthcare professionals, and researchers previously detailed elsewhere [1, 6].

The STAR trial recruited participants from eight NHS hospitals between September 2016 and May 2019. The study population were adults, aged 18 years or over who underwent a primary TKR due to osteoarthritis and who reported pain three months after the operation. Participants with a pain component score of the Oxford Knee Score (OKS-PS, scaled from 0 to 28) of 14 or less were classified as having chronic pain. Conversely, those with a score higher than 14 were classified as having no chronic pain [3]. The OKS was developed and validated for use by patients undergoing knee arthroplasty [10] and it is used as the primary disease-specific outcome measure within the NHS national PROMs Programme. It was found to have the highest (“good evidence in favour”) reproducibility, internal consistency, content and construct validity, responsiveness, and acceptability of all knee measures assessed in a systematic review of PROMs used in patients undergoing hip and knee arthroplasty [11]. Although the OKS assesses a single construct and is typically used as a unified measure, the pain subscale reports strong internal consistency allowing for its use as primary endpoint in clinical trials targeting pain improvement, as was the case with the STAR trial [12]. Further details of the demographics of trial participants and the exclusion criteria have been detailed previously [8]. The setting of this analysis was patients treated by the NHS in England and Wales at a hospital level of care. Only participants who provided informed written consent were included in the trial. Ethics approval was given by the South West-Central Bristol Research Ethics Committee (16/SW/0154). The STAR trial is registered with ISRCTN, ISRCTN92545361.

### Study perspective

The study was undertaken from the perspective of the NHS. As a result, only costs incurred by the NHS were considered in our analysis. The analysis was conducted using the willingness-to-pay threshold of £20,000 following the NICE guideline recommendation of £20,000-£30,000 [13].

### Decision model

**Fig. 1:**
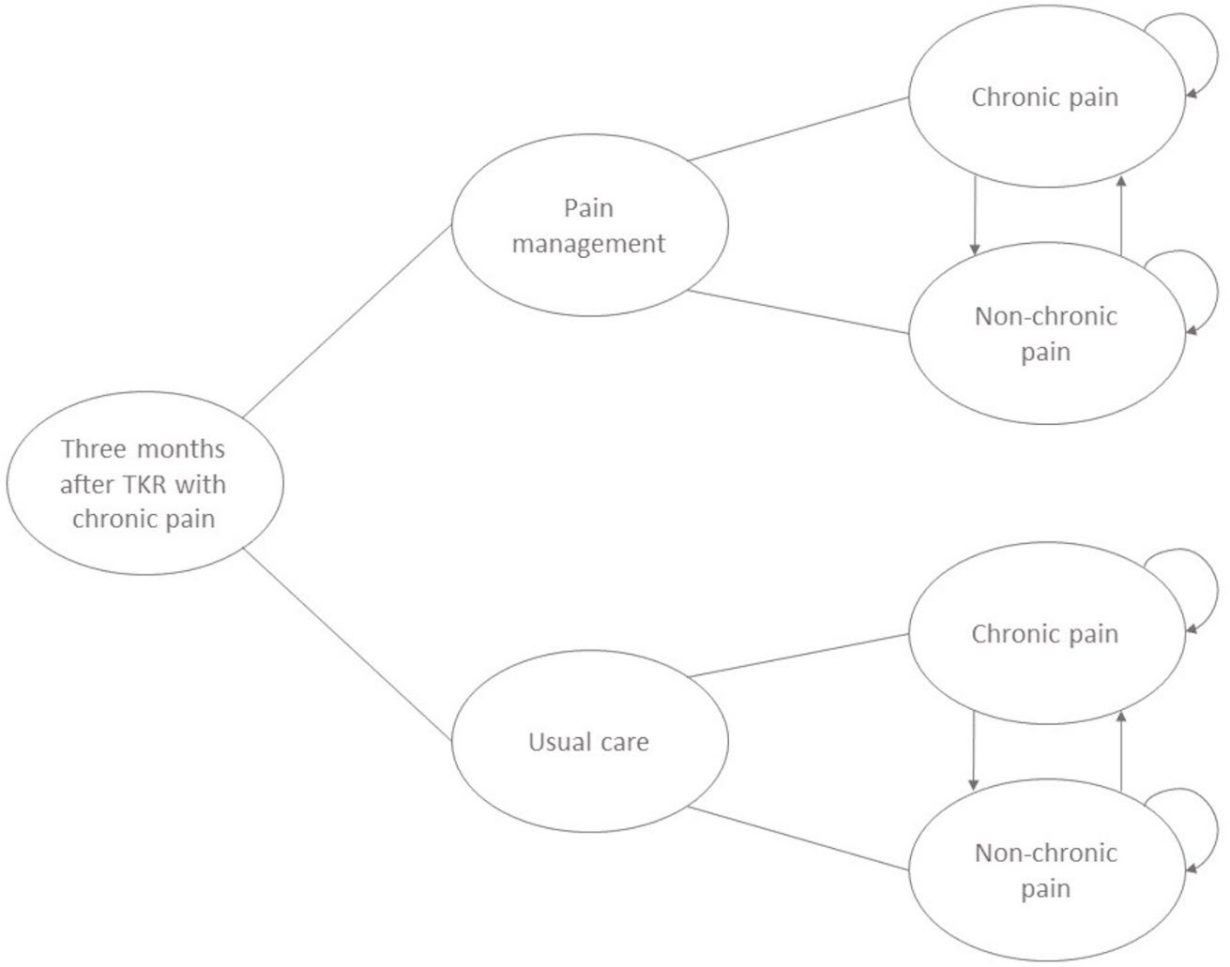
Model structure

The decision-analytic model was designed as a cohort Markov simulation with time-dependent annual transition probabilities. It was comprised of two health states, chronic pain (CP) and non-chronic pain (NCP), which followed either usual care or the STAR pain management intervention (see Fig. 1). Each cycle length was one year, and the time horizon was five years. Although shorter cycle lengths might more accurately capture the impact of changes in pain levels, the evidence available and used for this study to model the transition of patients through chronic pain states was collected on a yearly basis. This guided our choice for the yearly cycle length, as interpolating shorter interval transition probabilities would add potentially more bias than benefit from quicker transitions. As the STAR care pathway was developed with the objective of helping patients improve pain management by enabling referral to existing services based on the underlying reasons for chronic pain [6], a time horizon of five years was considered sufficient to capture the expected main impact of the intervention compared to usual postoperative care. Previous research has shown that most individuals who report CP one year after a TKR no longer experience it at the five-year follow-up point, thus suggesting that a time horizon of five years is sufficient and appropriate [14]. In the economic model, CP and NCP are classified using the same method (OKS-PS) as the STAR trial, detailed above. Health utility and costs were discounted from the second year onwards at 3.5% per year following NICE guidelines [15]. The development of the model was carried out as an iterative process by discussing the conceptualisation of the approach, structure, health states, transitions, assumptions, and data sources with the STAR Programme team which included surgeons, rheumatologists, pain specialists, epidemiologists, statisticians, qualitative researchers, and patient representatives. The final model was adopted once consensus was obtained that it represented pain states and progression appropriately and that it was fit for purpose, with an appropriate balance of simplicity and complexity so as to preserve face validity for both experts and patients.

All simulated patients who enter the model are classified as having CP at three months post-operation and were allocated either to receive usual postoperative care alongside the STAR care pathway (for the first year only) or to usual postoperative care only. At the end of the first cycle, simulated patients can then either transition to a NCP health state or remain in chronic pain. At any cycle thereafter patients can move from CP to NCP and vice versa. We did not include a health state of death in the model because the length of the time horizon was not sufficiently long and it was assumed that the STAR intervention does not influence the risk of mortality.

### Data

Model inputs were parameterised using evidence from a combination of three data sources (see Table 1 for values and sources for each model parameter). For the intervention and usual care comparator, data were collected from the STAR randomised control trial [16] with one year follow-up. For subsequent years (2 to 5), real-world evidence from COASt, a population-based cohort study with 5-year follow-up participant-reported data [17], and stand-alone longitudinal data extracted from the Clinical Practice Research Datalink (CPRD) were utilised alongside the STAR trial data. The COASt study recruited participants from two English NHS hospitals and their chronic pain status was categorised with the OKS-PS, measured yearly for five years. Participants were recruited from the knee replacement waiting list, regardless of age, gender, and body mass index. The CPRD sample consisted of 5,055 patients who underwent a TKR replacement surgery in England between 2009 and 2016 and were categorised as either having CP or NCP using the same OKS-PS threshold applied in the STAR trial.

**Table 1:** Input parameters

	Y1	Y2	Y3	Y4	Y5	Source
**Base case**						
*Transition probabilities*						
Usual care CP to CP	0.494	0.368	0.388	0.400	0.406	*STAR trial (Y1), * *COASt (Y2-Y5)*
Usual care CP to NCP	0.506	0.632	0.612	0.600	0.594	
Usual care NCP to CP	0.000	0.050	0.042	0.031	0.037	
Usual care NCP to NCP	0.000	0.950	0.958	0.969	0.963	
STAR care pathway CP to CP	0.355	0.368	0.388	0.400	0.406	
STAR care pathway CP to NCP	0.645	0.632	0.612	0.600	0.594	
STAR care pathway NCP to CP	0.000	0.050	0.042	0.031	0.037	
STAR care pathway NCP to NCP	0.000	0.950	0.958	0.969	0.963	
*Quality Adjusted Life Years*						
Usual care CP	0.465	0.556	0.607	0.642	0.698	*STAR trial (level),* * COASt (progression)*
Usual care NCP	0.539	0.730	0.727	0.716	0.702	
STAR care pathway CP	0.484	0.572	0.621	0.654	0.708	
STAR care pathway NCP	0.560	0.742	0.739	0.728	0.714	
*Cost (£)*						
STAR care pathway	191	0	0	0	0	*STAR trial*
Prescriptions CP	100	88	91	91	91	*STAR trial (level), * *CPRD (progression)*
Prescriptions NCP	22	18	17	17	18	
Medical consultations CP	166	143	137	137	125	*STAR trial (level),* * CPRD (progression)*
Medical consultations NCP	62	51	48	46	48	
Usual care hospital admissions CP	2,972	898	464	65	24	*STAR trial (level), * *COASt (progression)*
Usual care hospital admissions NCP	1,057	386	331	82	249	
STAR care pathway hospital admissions CP	1,265	898	464	65	24	*STAR trial (level), * *COASt (progression)*
STAR care pathway hospital admissions NCP	1,302	386	331	82	249	
**Sensitivity analysis - scenario analysis**						
*Quality Adjusted Life Years– Scenario 1*						
CP	0.477	0.566	0.616	0.650	0.704	*STAR trial (level), * *COASt (progression)*
NCP	0.555	0.739	0.736	0.725	0.711	
*Transition probabilities– Scenario 2*						
Usual care CP to CP	0.500	0.368	0.388	0.400	0.406	*STAR trial (Y1),* * COASt (Y2-Y5)*
Usual care CP to NCP	0.500	0.632	0.612	0.600	0.594	
Usual care NCP to CP	0.000	0.050	0.042	0.031	0.037	
Usual care NCP to NCP	1.000	0.950	0.958	0.969	0.963	
STAR care pathway CP to CP	0.357	0.368	0.388	0.400	0.406	
STAR care pathway CP to NCP	0.643	0.632	0.612	0.600	0.594	
STAR care pathway NCP to CP	0.222	0.050	0.042	0.031	0.037	
STAR care pathway NCP to NCP	0.778	0.950	0.958	0.969	0.963	
*Cost (£)– Scenario 3*						
Hospital admissions CP	1,935	898	464	65	24	*STAR trial (level), * *COASt (progression)*
Hospital admissions NCP	1,237	386	331	82	249	

Level - refers to the data used to estimate the initial year one input parameters

Progression - refers to the data used to estimate the change over time which was then applied to the initial year one input parameters to estimate subsequent years

### Transition probabilities

We estimated transition probabilities for moving from CP at baseline to either CP or NCP in year 1 using evidence from the STAR trial as it is during the first cycle that the STAR care pathway was provided. These transition probabilities were taken separately for the intervention and control arms of the trial, leading hence to different probabilities for the STAR care pathway and usual care comparators. For all subsequent cycles (years two to five) the transition probabilities between CP and NCP in either direction were estimated using evidence from COASt and applied equally to both comparators. This represents a conservative assumption that improvements in pain management because of the STAR care pathway would be experienced only whilst the intervention is active, and the trial only applied the intervention during the first 12 months. Any benefits of moving out of chronic pain during the first year, however, would still be enjoyed by all simulated patients managing to move from CP to NCP during that first year. Further details about the sources and calculation of transition probabilities are provided in the supplementary material.

### Costs

Our cost parameter estimates were calculated considering a combination of the cost of the STAR intervention, primary care consultations, medication prescriptions, and hospital admissions. For the first cycle, costs were estimated using data from the respective trial arms. For all subsequent years (2 to 5), we used a combination of real-world evidence from CPRD (prescriptions and consultations) and COASt (hospital admissions) data. We calculated the annual percentage changes in costs (prescriptions, consultations, and hospital admissions) observed for CP and NCP groups respectively and applied them appropriately to their respective year-one trial estimates. Costs from CPRD comprised of all detailed pain prescriptions and primary care consultations, but costs from the STAR trial and COASt cohort comprised of only costs related to treatment of the knee and the pain associated to the TKR. Based upon the evidence from the trial, primary care consultations, medication prescriptions, and hospital admission costs differed between arms. The model was populated with different hospital costs for comparators during the first cycle only, with costs during the following cycles being estimated from the trial’s 12-month hospital admissions cost for NCP and CP (difference by arm no longer considered) multiplied by the appropriate annual percentage changes observed in the COASt data over five years. For the STAR intervention comparator, there was an additional cost of £191 for the intervention comprising of costs associated with the STAR care pathway such as the clinical assessment and any subsequent follow-up phone calls, which was applied in the first cycle only. Further information about the costing of the intervention were detailed previously [8]. Costs for CP and NCP health states were calculated independently and estimated in pound sterling (£) using 2019–2020 prices. Further details about the sources and calculation of costs are provided in the supplementary material.

### Health utility estimates

Health utility estimates for the first cycle were calculated using the reported EQ-5D-5 L descriptive responses mapped onto the EQ-5D-3 L value set using the mapping function [18] recommended by NICE. Although there is a published EQ‑5D‑5 L value set for England [19], NICE recommends not using it due to reported concerns about the quality and reliability of the data used in the study as well as the methods applied, and to use the mapping function instead [20]. We used the area under the curve method to estimate mean quality-adjusted life years (QALYs) using the baseline, 6-months’ and 1-year’s health utility estimates from the STAR trial. The QALY estimates were subsequently adjusted using multiple regression to control for baseline health utility [21]. This means that, although all theoretical patients with CP (or NCP) enter the model having the same level of health utility regardless of the pathway they follow, the QALYs accrued during year 1 for the same health state vary depending on the pathway they follow as reported in the trial. Differences in QALYs during the first year for the same health state between the different pathways reflect the findings of the trial for the two arms separately. This approach was changed under a sensitivity analysis.

For all years following (2 to 5), QALY values were calculated by taking the trial QALY estimates and applying the yearly change observed in COASt for each group, CP and NCP as classified at 12-months. The yearly change observed in COASt was measured using the percentage of potential change (PoPC) method [22], which calculates change as a percentage of the potential change possible. See supplementary material for further details.

### Analysis

We generated summary statistics of patient characteristics of the three main sources of data one year post-operatively to assess the comparability of the data sources.

### Base case analysis

According to trial results, for both comparators (the STAR care pathway and usual care) the QALY estimates were different between the two health states, CP and NCP, with CP being lower than NCP for both arms. Individuals were identified as having CP at three months (baseline) after the knee replacement in the base case. To assess the longer-term cost-effectiveness of the intervention, we calculated the expected benefits (gains in QALYs) and expected costs of the two comparators and calculated the incremental net monetary benefit (iNMB) of the STAR pathway. Additionally, we reported a 95% confidence interval ellipse for the base case cost-effectiveness plane. The incremental cost-effectiveness ratio (ICER) was calculated when clear dominance was not displayed by either comparator.

### Scenario analyses

Scenario analysis was used to assess the effect on model results of varying assumptions made based on evidence found in the trial.

#### Scenario 1

Firstly, we tested the removal of the difference between the QALY estimates for the STAR care pathway and usual care for both CP and NCP. This was implemented to test the assumption made in the base case analysis that the health states (CP and NCP) QALY estimates would differ by comparator, as found in the trial. As a result, each CP and NCP health states had a single QALY estimate regardless of the comparator, which was introduced in the first cycle. As PoPC was applied to generate corresponding values for CP and NCP for all following cycles (years 2 to 5), QALY estimates for the CP and NCP health states were the same across the comparators. Transition probabilities remained the same as the base case, i.e. they differed between arms, hence each strategy could accrue a different total number of QALYs based solely on how simulated patients transitioned through the CP and NCP health states. These results were then compared to the base case to assess how the assumption impacted the model.

#### Scenario 2

The base case assumes that the intervention starts at 12 weeks post-operation and is applied to people who have CP. In the trial, however, although the intervention started at 12 weeks post-operation, the initial CP status was assessed at 10 weeks. This meant that two weeks later, at baseline, some patients could have moved out of CP. This scenario considers this and allows for some people to enter the model classified with NCP. Again, results from this scenario analysis were compared to base case results.

#### Scenario 3

For our third scenario we considered hospital costs differing not by comparator but only by health states (CP and NCP) and for the first cycle. This meant that changes in costs would be driven by the ability of the STAR intervention to move people in or out of CP, assuming hence that someone with or without CP would use the same level of healthcare resources whether they get the intervention or not (aside from the direct costs of the intervention itself). Hospital costs for all subsequent cycles (years 2 to 5) were estimated following the same approach as the base case. We then compared the results of this scenario with the base case.

### Probabilistic sensitivity analysis (PSA)

We tested the uncertainty of the model parameters and their effect on the model results by applying appropriate distributions to transition probabilities, costs, and health utilities. The model was then run for 10,000 independent simulations. A cost-effectiveness acceptability curve (CEAC) was generated from the simulated results to report the probability of cost-effectiveness considering the uncertainty of the model input parameters.

The economic evaluation is reported following the CHEERS checklist [23]. No model-based health economic analysis plan was developed prior to the completion of this analysis and the model is not publicly available. All analyses were completed using the computer software R version 4.0.3 (2020-10-10) [24] and additional packages were utilised to complete data cleaning, analysis, and to generate all figures [25–27].

## Results

Mean population age across the three sources of data ranged from 68 to70 years old (see Table A.1 in the supplementary material). STAR reported a lower percentage of male participants than COASt and CPRD-HES with 34%, 44% and 44%, respectively. Mean scores of EQ-5D-3 L of the total sample were lower for STAR than CPRD-HES and COASt data with mean scores of 0.556, 0.740, and 0.741, respectively. Mean scores of OKS for STAR were also lower than COASt and CPRD-HES (see Table A.1). Participants in the STAR intervention arm of the trial moved out of the chronic pain state at a faster rate than those who received usual care only. This is shown in Figure A.1 where 46% of those who received the STAR intervention were still in chronic pain at six months compared to 54% for those who received usual care. Input parameters and their probability sensitivity analysis distributions for the economic model are reported in Table 1 and in the supplementary material (Tables A.2a-b).

**Fig. 2:**
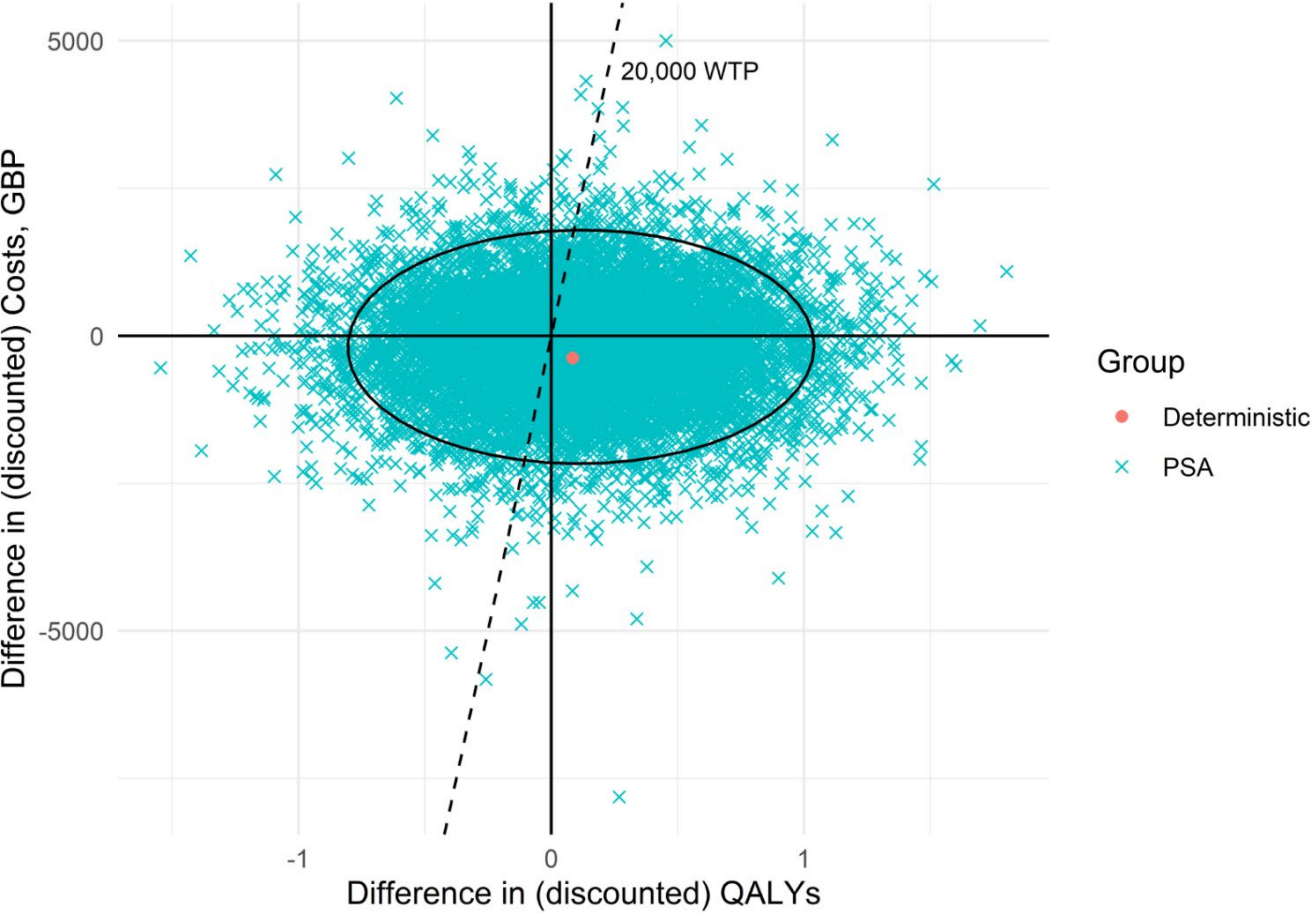
Base case cost-effectiveness plane

Results of the base case analysis show that the STAR care pathway would lead to savings of £375 per patient compared to usual care (£3,563 vs. £3,189, respectively), and achieve 0.086 more QALYs (3.091 vs. 3.177) over the five years. This suggests that the STAR intervention is dominant over usual care. The incremental net monetary benefit (iNMB) of the STAR intervention was £2,086 (confidence interval [-£14,234, £19,644]) at a cost-effectiveness threshold of £20,000 per QALY gained (see Table 2). Results of the probability sensitivity analysis (PSA) show that the STAR intervention has a probability of 0.62 of being cost-effective at said threshold (see Fig. 2).

**Table 2:** Results from the base case model and scenario analysis

	STAR intervention		Usual care		Difference			
	QALYs	Costs £	QALYs	Costs £	∆ QALYs	∆ Costs £	iNMB*	Probability STAR intervention is cost-effective (PSA*)
Base case	3.18	3188.86	3.09	3563.45	0.086	-374.59	2086.09	0.62
Scenario 1 (QALY varies by CP/NCP and not by comparator)	3.16	3188.86	3.14	3563.45	0.020	-374.59	781.55	0.64
Scenario 2 (CP classification at 10 weeks)	3.18	3185.06	3.10	3476.38	0.082	-291.32	1924.61	0.61
Scenario 3 (No difference in hospital costs at year 1)	3.18	3141.79	3.09	3141.79	0.086	242.91	1468.59	0.59

*iNMB and PSA analysis calculated with a threshold of £20,000

STAR, Support and Treatment After Replacement; QALYs, quality-adjusted life years; iNMB, incremental net monetary benefit; PSA, probability sensitivity analysis; CP, chronic pain; NCP, non-chronic pain

### Scenario 1

When we assumed that the QALY estimated for the two health states were identical between the two comparators, the model reported that the STAR intervention remained dominant over usual care in the deterministic analysis with an iNMB of £782 (see Table 2) and a probability of 0.64 of being cost-effective from the PSA analysis (see Figure A.2 in the supplementary material).

### Scenario 2

The STAR care pathway remained deterministically dominant compared to usual care when the CP status was classified at 10 weeks after a TKR (instead of 12 weeks as used in the base case) with an iNMB of £1,925 (see Table 2). The probability sensitivity analysis found that the STAR intervention had a cost-effectiveness probability of 0.61 (see Figure A.3).

### Scenario 3

Lastly, when we assumed that the hospital admission costs were the same for both pain health states regardless of comparator (STAR care pathway and usual care), the STAR intervention still reported higher QALY estimates than usual care (3.18 vs. 3.09) but costs became higher for the STAR intervention (£243, see Table 2). This scenario reported a deterministic iNMB of £1,469, an incremental cost-effectiveness ratio of £2,839 per QALY gained, and a cost-effectiveness probability of 0.59 (see Figure A.4 in the supplementary material).

## Discussion

Evidence from the economic model indicates that the STAR intervention when compared with usual care only is expected not only to be more effective but also cost-saving for the first five years after TKR. This result supports the within-trial analysis which found that the STAR care pathway was cost-effective 12 months after the TKR [8, 9]. Our economic model, however, found the average cost-savings per patient (£375 over 5 years) to be lower than that found in the within-trial analysis (£724). This is likely due to differences in how costs are calculated in the within-trial analysis compared to the modelling. Whereas for the within-trial analysis the costs of every participant in each arm are pooled and summarised, for the modelling we classified people by their expected pain status and used that to assign them to corresponding health states which had specific costs associated to them. The modelling will tend to approximate findings from the trial, but as the model covered five years, the classification of simulated patients at every cycle into CP status and their corresponding assignment of costs is likely to move cost-saving estimates further away from what was found in the trial.

The improvement in QALYs found was small, and below the minimally clinically important difference of 0.182 for knee replacement surgery [28]. Probabilistic sensitivity analysis found that the STAR care pathway is likely to be cost-effective. Nonetheless, there is a considerable amount of uncertainty surrounding this result, illustrated by the 95% confidence interval ellipse which crosses all four quadrants of the cost-effectiveness plane (Fig. 2). This suggests that either comparator, the STAR intervention or usual care, could potentially provide QALY gains or increase in costs.

Testing of the model assumptions of equal comparator health utility and the timing of the intervention suggest that the results are robust, with the STAR intervention remaining dominant. When CP is classified at 10 weeks post-operation (compared to 12 weeks), the model shows that this is a factor which impacts the cost-effectiveness of the model. The results suggest that the STAR intervention is more cost-effective when CP is categorised at 12 weeks post-operation and may indicate that it would be preferable to wait those two weeks before assessing patients’ CP status and considering the STAR care pathway. This improvement in cost-effectiveness, however, is minimal. The STAR care pathway’s dominance was not robust to removal of the intervention’s effect on hospital admission costs and showed it was a clear factor in the cost-saving base case results. The scenario results showed that the STAR care pathway nonetheless be expected to be highly cost-effective.

As TKRs are commonly performed in the UK, the improvement of the patient care pathway with inclusion of the STAR intervention has the potential to provide a large positive impact despite the small improvements in health-related quality of life. Based on the approximately 100,000 TKR surgeries that are performed each year and assuming that up to 20% of patients experience chronic pain, a national rollout of the STAR intervention with effectiveness in its real-world application similar to what was shown in the trial could potentially lead to cost savings in healthcare (intervention, primary care consultations, medication prescriptions, and hospital admissions) of around £7.5 million over five years for each annual cohort of patients exposed. This excludes potential savings in social care costs, which fell outside the scope of this study, and would be accompanied by a corresponding improvement in patients’ quality of life as well as potential other benefits to their families, carers, and wider productivity.

The trial recruited participants from multiple hospitals across England and Wales which adds to the generalisability of results. However, the lack of inclusion of hospitals from other parts of the UK may limit its generalisability to the wider UK population. The economic evaluation was conducted from the perspective of the NHS and as such this may also limit the generalisability of its findings to other countries where the health care system is not universal or differs widely from that of England and Wales.

An important strength of this study is that it estimates the value and the impact of the STAR intervention over a longer five-year time horizon compared to the 12-month window assessed during the trial. This provides a fuller picture of the post-surgical experience of people with chronic pain following a TKR. Additionally, our analysis considered the potential scenario of hospital costs being determined only by whether people are in CP or not, removing the differences found in the trial about hospital costs for people in CP or non-CP depending on whether they have the intervention or not. Exploring this allowed this study to provide evidence that, even if that were the case, the STAR intervention would be expected to cease being dominant but still achieve high cost-effectiveness with an ICER of £2,839 per QALY gained.

A limitation of this economic evaluation was that the model was populated with data from three different sources each of which classified CP at different time points. Evidence from the trial found that those who received the STAR intervention moved out of the CP health state at a faster rate and earlier than those who received only usual care at the six-month time point. As the cycle length was one year, this earlier transition by those who received the STAR intervention was not captured in the economic model. This may have led to an underestimation of the QALYs gain and an overestimation of costs for the STAR intervention comparator.

A further limitation of the study was that the comparison of the sample data showed some differences in the population characteristics such as EQ-5D-3 L. However, these differences can be attributed to the sample make-up; e.g. only those categorised with CP were included in STAR and the time point at which measurements were taken differed from those of the COASt cohort. We mitigated these sample differences for health utility by implementing the PoPC method which applied the annual change from the COASt data to the STAR trial health utility estimates, thereby anchoring levels at what was found in the trial but looking at the COASt cohort for changes over time. Unfortunately, further common and relevant patient characteristics (e.g. socio-economic background) were not available to adjust for any further differences in sample demographics, however including data from a trial and from real-world sources helped produced a more balanced analysis than if either alone had been used. Nevertheless, an external validation of this model using data from other research studies on interventions for the management of chronic pain in patients undergoing total knee replacement is warranted.

## Conclusions

The STAR care pathway modelled from an NHS perspective over five years after TKR is likely to be cost-effective. The simulation found the benefits of increased health-related quality of life to be modest but robust to scenarios reflecting greatest uncertainty of inputs, with potential reduction in costs conditional on reduced hospital admissions after the operation for those in the STAR intervention. Further research into the long-term follow-up of trial participants is currently being undertaken; findings from that work will help validate some assumptions and aid in the reduction of uncertainty of the economic model results.

### Electronic supplementary material

Below is the link to the electronic supplementary material.

Supplementary Material 1

## Data Availability

The STAR trial data sets used during the present study are available from the University of Bristol Research Data Repository. Access is subject to requirements. Analysed CPRD data are available from the corresponding author, but restrictions apply to its availability as they were used under license for this specific analysis and are not publicly available. COASt data are also available from the corresponding author, but access is subject to researchers submitting a completed application to the COASt Data and Sample Access Committee.

## Abbreviations

CEAC: cost-effectiveness acceptability curve
COASt: Clinical Outcomes in Arthroplasty Study
CPRD: Clinical Practice Research Datalink
CP: chronic pain
ICER: incremental cost-effectiveness ratio
iNMB: incremental net monetary benefit
NCP: non-chronic pain
NHS: National Health Service
OKS-PS: Oxford Knee Score Pain Subscale
PoPC: percentage of potential change
PSA: probability sensitivity analysis
QALYs: quality-adjusted life years
STAR: Support and Treatment After joint Replacement
TKR: total knee replacement
