# Supplementary material for: Modelled cost-effectiveness analysis of the Support and Treatment After Replacement (STAR) care pathway for chronic pain after total knee replacement compared with usual care
Source: Cost Eff Resour Alloc. 2024 Apr 11;22:28. doi: 10.1186/s12962-024-00532-5 (PMC11010279; doi:10.1186/s12962-024-00532-5)
Supplement: Supplementary file 1 — Supplementary Material 1 [file 12962_2024_532_MOESM1_ESM.docx]

**Supplementary material – online only**

**Additional methods**

Transition probabilities

The first year transition probabilities were estimated using the STAR trial data. The COASt study measured chronic pain (CP) at five follow-up time points (each a year apart). We utilised the COASt data to calculate separate transition probabilities for each subsequent year (Y2-5) of the model. The uncertainty of the transition probabilities were modelled using a beta distribution (see supplementary material table A.1).

Costs

STAR trial reported resource use was costed using a combination of NHS reference costs and Personal Social Services Research Unit costs which was detailed previously (1). To generate hospital cost estimates for the years 2-5 we used the reported costs for each arm (estimated from the STAR trial data) at year one and applied the change observed in hospital costs (e.g. from year one to year two, etc.) reported in the COASt data. More information on how the hospital resource use was costed has been detailed in previous research (2). Both consultation and prescriptions costs for years two to five were estimated by measuring the change in costs over time. For this we used Clinical Practice Research Datalink (CPRD) data for a sample of 721 individuals who reported CP following a TKR and 4334 individuals who reported non-chronic pain (NCP) following a TKR. These changes overtime in consultation and prescription costs reported in the CPRD data split by CP/NCP status were then applied to their equivalent year one costs reported in the STAR trial to generate input parameter costs for years two to five. The uncertainty of the costs were modelled using a gamma distribution (see supplementary material table A.1).

Health utilities

Previous evidence from the COASt study found that those with CP following a TKR improved slowly but steadily overtime (2). Based on this evidence we applied the same utility pathway in our economic model. To acknowledge and account for differences in study populations between the STAR trial and the COASt study we applied the percentage of potential change (PoPC) measured in the COASt study to the level of utility measured in the START trial (3). This was to ensure that the economic model reflected the utility values measured in the STAR trial population. This meant that the PoPC in utility measured from CP year one to CP in year two was then applied to the original STAR trial CP year one to generate the year two CP utility model estimate. The same process was followed to calculate the year two NCP utility model estimate. All subsequent years were calculated accordingly. The uncertainty of the utility values were modelled using a beta distribution (see supplementary material table A.1).

**References**

1. Wylde V, Bertram W, Sanderson E, Noble S, Howells N, Peters TJ, et al. The STAR care pathway for patients with pain at 3 months after total knee replacement: a multicentre, pragmatic, randomised, controlled trial. 2022.
2. Cole S, Kolovos S, Soni A, Delmestri A, Sanchez-Santos MT, Judge A, Arden NK, Beswick AD, Wylde V, Gooberman-Hill R, Pinedo-Villanueva R. Progression of chronic pain and associated health-related quality of life and healthcare resource use over 5 years after total knee replacement: evidence from a cohort study. BMJ Open. 2022 Apr 25;12(4):e058044.
3. Kiran A, Bottomley N, Biant LC, Javaid MK, Carr AJ, Cooper C, et al. Variations In Good Patient Reported Outcomes After Total Knee Arthroplasty. J Arthroplasty. 2015;30(8):1364-71.

**Tables**

| Table A.1: A comparison of data sample population categorised in chronic pain at year 1 | | | | | | | | | | |
| --- | --- | --- | --- | --- | --- | --- | --- | --- | --- | --- |
| Study name | | CP | | | NCP | | | Total | | |
|  |  | n | mean | SD | n | mean | SD | n | mean | SD |
| Age | STAR | 42 | 67 | 9.44 | 50 | 69 | 8.65 | 92 | 68 | 9.06 |
|  | COASt | 70 | 70 | 8.58 | 482 | 70 | 8.61 | 552 | 70 | 8.60 |
|  | CPRD-HES | 721 | 67 | 9.69 | 4334 | 69 | 8.63 | 5055 | 69 | 8.84 |
| OKS-PS | STAR | 42 | 10.19 | 2.62 | 50 | 20.88 | 3.49 | 92 | 16.00 | 6.19 |
|  | COASt | 70 | 9.66 | 3.59 | 482 | 23.84 | 3.97 | 552 | 22.04 | 6.14 |
| OKS | STAR | 42 | 18.33 | 4.48 | 50 | 34.54 | 6.16 | 92 | 27.14 | 9.77 |
|  | COASt | 67 | 16.87 | 6.02 | 472 | 38.90 | 6.94 | 539 | 36.16 | 9.98 |
|  | CPRD-HES | 721 | 18.06 | 5.52 | 4334 | 38.20 | 6.67 | 5055 | 35.32 | 9.59 |
| EQ-5D-3L | STAR | 42 | 0.403 | 0.215 | 50 | 0.685 | 0.169 | 92 | 0.556 | 0.237 |
|  | COASt | 67 | 0.386 | 0.310 | 471 | 0.791 | 0.199 | 538 | 0.740 | 0.254 |
|  | CPRD-HES | 628 | 0.417 | 0.293 | 3828 | 0.795 | 0.192 | 4456 | 0.741 | 0.247 |
|  |  |  | CP % | | NCP % | | | Total % | | |
| Sex | STAR | M | 31% | | 36% | | | 34% | | |
|  |  | F | 69% | | 64% | | | 66% | | |
|  | COASt | M | 31% | | 46% | | | 44% | | |
|  |  | F | 69% | | 54% | | | 56% | | |
|  | CPRD-HES | M | 42% | | 44% | | | 44% | | |
|  |  | F | 58% | | 56% | | | 56% | | |
| *For STAR year one outcomes we considered the control group only (usual care)* | | | | | | | | | | |

Table A.2a - Base case parameter distributions

| Transition probabilities | | | | | | | | |
| --- | --- | --- | --- | --- | --- | --- | --- | --- |
|  | Non-CP to Non-CP | | | CP to Non-CP | | |  | Parameters for  the distribution |
| Usual care | Prob | α | β | Prob | α | β | Distribution |  |
| Y1 | 0.000 | 0 | 0 | 0.506 | 43 | 42 | Beta | α=events β=sample size - α |
| Y2 | 0.950 | 458 | 24 | 0.632 | 43 | 25 |  |  |
| Y3 | 0.958 | 480 | 21 | 0.612 | 30 | 19 |  |  |
| Y4 | 0.969 | 494 | 16 | 0.600 | 24 | 16 |  |  |
| Y5 | 0.963 | 499 | 19 | 0.594 | 19 | 13 |  |  |
| STAR care pathway | Prob | α | β | Prob | α | β |  |  |
| Y1 | 0.000 | 0 | 0 | 0.645 | 118 | 65 | Beta | α=events β=sample size - α |
| Y2 | 0.950 | 458 | 24 | 0.632 | 43 | 25 |  |  |
| Y3 | 0.958 | 480 | 21 | 0.612 | 30 | 19 |  |  |
| Y4 | 0.969 | 494 | 16 | 0.600 | 24 | 16 |  |  |
| Y5 | 0.963 | 499 | 19 | 0.594 | 19 | 13 |  |  |
|  | Non-CP to CP | | | CP to CP | | |  |  |
| Usual care | Prob | α | β | Prob | α | β |  |  |
| Y1 | 0.000 | 0 | 0 | 0.494 | 42 | 43 | Beta | α=events β=sample size - α |
| Y2 | 0.050 | 24 | 458 | 0.368 | 25 | 43 |  |  |
| Y3 | 0.042 | 21 | 480 | 0.388 | 19 | 30 |  |  |
| Y4 | 0.031 | 16 | 494 | 0.400 | 16 | 24 |  |  |
| Y5 | 0.037 | 19 | 499 | 0.406 | 13 | 19 |  |  |
| STAR care pathway | Prob | α | β | Prob | α | β |  |  |
| Y1 | 0.000 | 0 | 0 | 0.355 | 65 | 118 | Beta | α=events β=sample size - α |
| Y2 | 0.050 | 24 | 458 | 0.368 | 25 | 43 |  |  |
| Y3 | 0.042 | 21 | 480 | 0.388 | 19 | 30 |  |  |
| Y4 | 0.031 | 16 | 494 | 0.400 | 16 | 24 |  |  |
| Y5 | 0.037 | 19 | 499 | 0.406 | 13 | 19 |  |  |
| Quality Adjusted Life Years | | | | | | | | |
|  | Non-CP | | | CP | | |  |  |
| Usual care | Mean | α | β | Mean | α | β | Distribution |  |
| Y1 | 0.539 | 7.58 | 6.48 | 0.465 | 5.69 | 6.54 | Beta | α=mean*(mean*(1-mean)/(SE^2)-1) β=α*(1-mean)/mean |
| Y2 | 0.730 | 0.81 | 0.30 | 0.556 | 0.23 | 0.19 |  |  |
| Y3 | 0.727 | 0.80 | 0.30 | 0.607 | 0.25 | 0.16 |  |  |
| Y4 | 0.716 | 0.82 | 0.33 | 0.642 | 0.25 | 0.14 |  |  |
| Y5 | 0.702 | 0.81 | 0.34 | 0.698 | 0.20 | 0.09 |  |  |
| STAR care pathway | Mean | α | β | Mean | α | β |  |  |
| Y1 | 0.560 | 11.23 | 8.83 | 0.484 | 6.12 | 6.53 | Beta | α=mean*(mean*(1-mean)/(SE^2)-1) β=α*(1-mean)/mean |
| Y2 | 0.742 | 0.77 | 0.27 | 0.572 | 0.23 | 0.18 |  |  |
| Y3 | 0.739 | 0.76 | 0.27 | 0.621 | 0.24 | 0.15 |  |  |
| Y4 | 0.728 | 0.79 | 0.30 | 0.654 | 0.24 | 0.13 |  |  |
| Y5 | 0.714 | 0.79 | 0.32 | 0.708 | 0.18 | 0.08 |  |  |

Table A.2b - Base case parameter distributions

| Costs | | | | | | | | | | |
| --- | --- | --- | --- | --- | --- | --- | --- | --- | --- | --- |
|  | Non-CP | | | | CP | | | |  | Parameters for the distribution |
| Consultations | Mean | SE | α | β | Mean | SE | α | β | Distribution |  |
| Y1 | 61.53 | 21.47 | 8.21 | 7.49 | 166.12 | 51.26 | 10.50 | 15.82 | Gamma | α <- (mean/SE)^2 β <- SE^2/mean |
| Y2 | 50.66 | 21.47 | 5.57 | 9.10 | 142.55 | 51.26 | 7.73 | 18.43 |  |  |
| Y3 | 47.84 | 21.47 | 4.96 | 9.64 | 137.29 | 51.26 | 7.17 | 19.14 |  |  |
| Y4 | 46.10 | 21.47 | 4.61 | 10.00 | 137.16 | 51.26 | 7.16 | 19.16 |  |  |
| Y5 | 47.66 | 21.47 | 4.93 | 9.67 | 125.29 | 51.26 | 5.97 | 20.97 |  |  |
|  | Non-CP | | | | CP | | | |  |  |
| Prescriptions | Mean | SE | α | β | Mean | SE | α | β |  |  |
| Y1 | 22.36 | 4.41 | 25.70 | 0.87 | 100.09 | 17.02 | 34.59 | 2.89 | Gamma | α <- (mean/SE)^2 β <- SE^2/mean |
| Y2 | 17.70 | 4.41 | 16.11 | 1.10 | 88.12 | 17.02 | 26.81 | 3.29 |  |  |
| Y3 | 17.02 | 4.41 | 14.91 | 1.14 | 91.10 | 17.02 | 28.66 | 3.18 |  |  |
| Y4 | 17.29 | 4.41 | 15.38 | 1.12 | 91.33 | 17.02 | 28.80 | 3.17 |  |  |
| Y5 | 17.95 | 4.41 | 16.56 | 1.08 | 91.47 | 17.02 | 28.89 | 3.17 |  |  |
| Hospital admissions | | | | | | | | | | |
|  | Non-CP | | | | CP | | | |  |  |
| Usual care | Mean | SE | α | β | Mean | SE | α | β | Distribution |  |
| Y1 | 1056.97 | 282.77 | 13.97 | 75.65 | 2972.36 | 780.42 | 14.51 | 204.90 | Gamma | α <- (mean/SE)^2 β <- SE^2/mean |
| Y2 | 386.42 | 179.70 | 4.62 | 83.56 | 897.53 | 344.55 | 6.79 | 132.27 |  |  |
| Y3 | 330.66 | 179.70 | 3.39 | 97.65 | 463.69 | 344.55 | 1.81 | 256.02 |  |  |
| Y4 | 82.13 | 179.70 | 0.21 | 393.15 | 65.16 | 344.55 | 0.04 | 1821.97 |  |  |
| Y5 | 249.40 | 179.70 | 1.93 | 129.47 | 24.35 | 344.55 | 0.00 | 4875.49 |  |  |
| STAR care pathway | Mean | SE | α | β | Mean | SE | α | β |  |  |
| Y1 | 1302.24 | 222.83 | 34.15 | 38.13 | 1264.65 | 231.67 | 29.80 | 42.44 | Gamma | α <- (mean/SE)^2 β <- SE^2/mean |
| Y2 | 386.42 | 179.70 | 4.62 | 83.56 | 897.53 | 344.55 | 6.79 | 132.27 |  |  |
| Y3 | 330.66 | 179.70 | 3.39 | 97.65 | 463.69 | 344.55 | 1.81 | 256.02 |  |  |
| Y4 | 82.13 | 179.70 | 0.21 | 393.15 | 65.16 | 344.55 | 0.04 | 1821.97 |  |  |
| Y5 | 249.40 | 179.70 | 1.93 | 129.47 | 24.35 | 344.55 | 0.00 | 4875.49 |  |  |

**Figures**

Figure A.1. Proportion in chronic pain


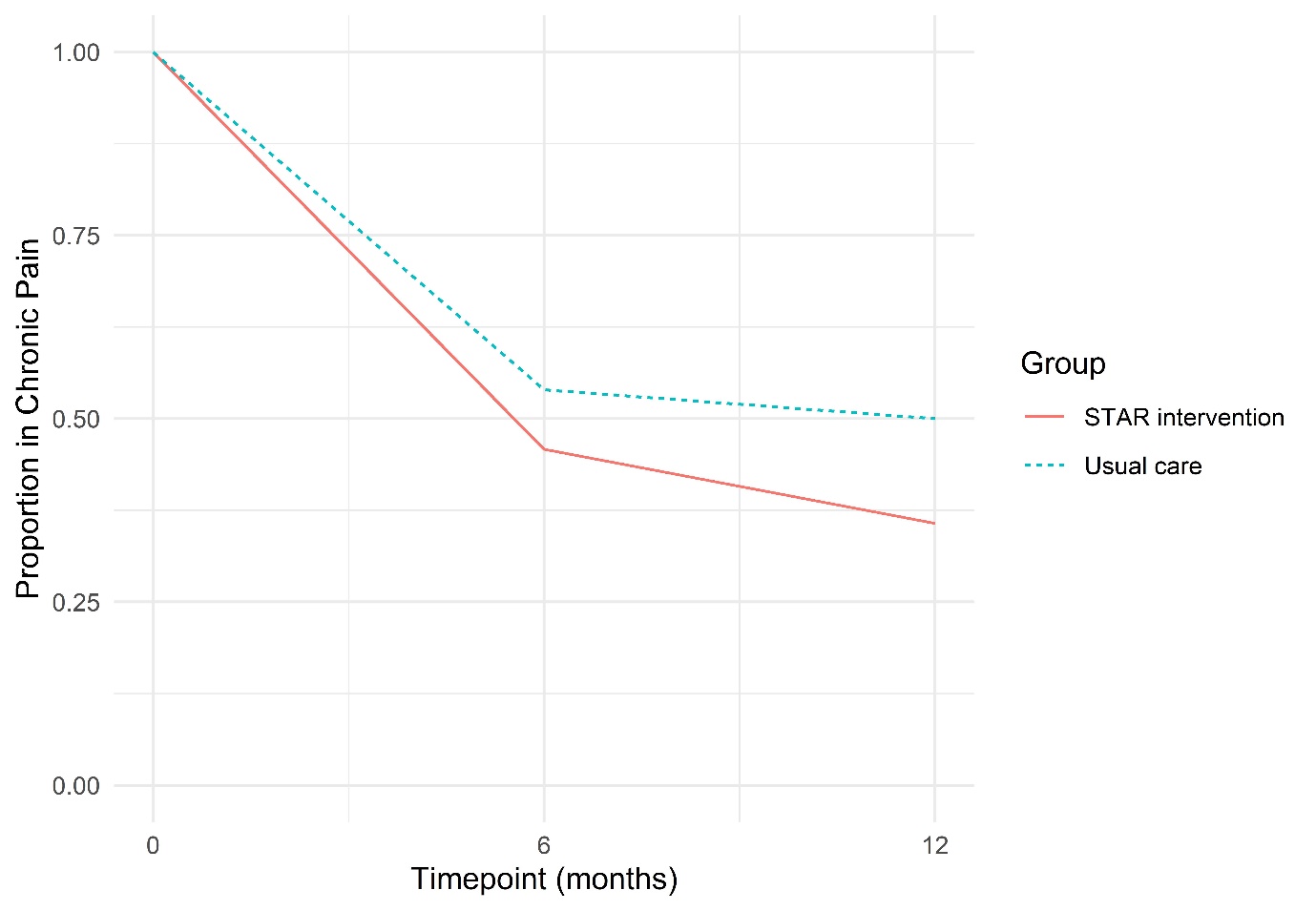


Figure A.2 Cost-effectiveness plane - scenario 1


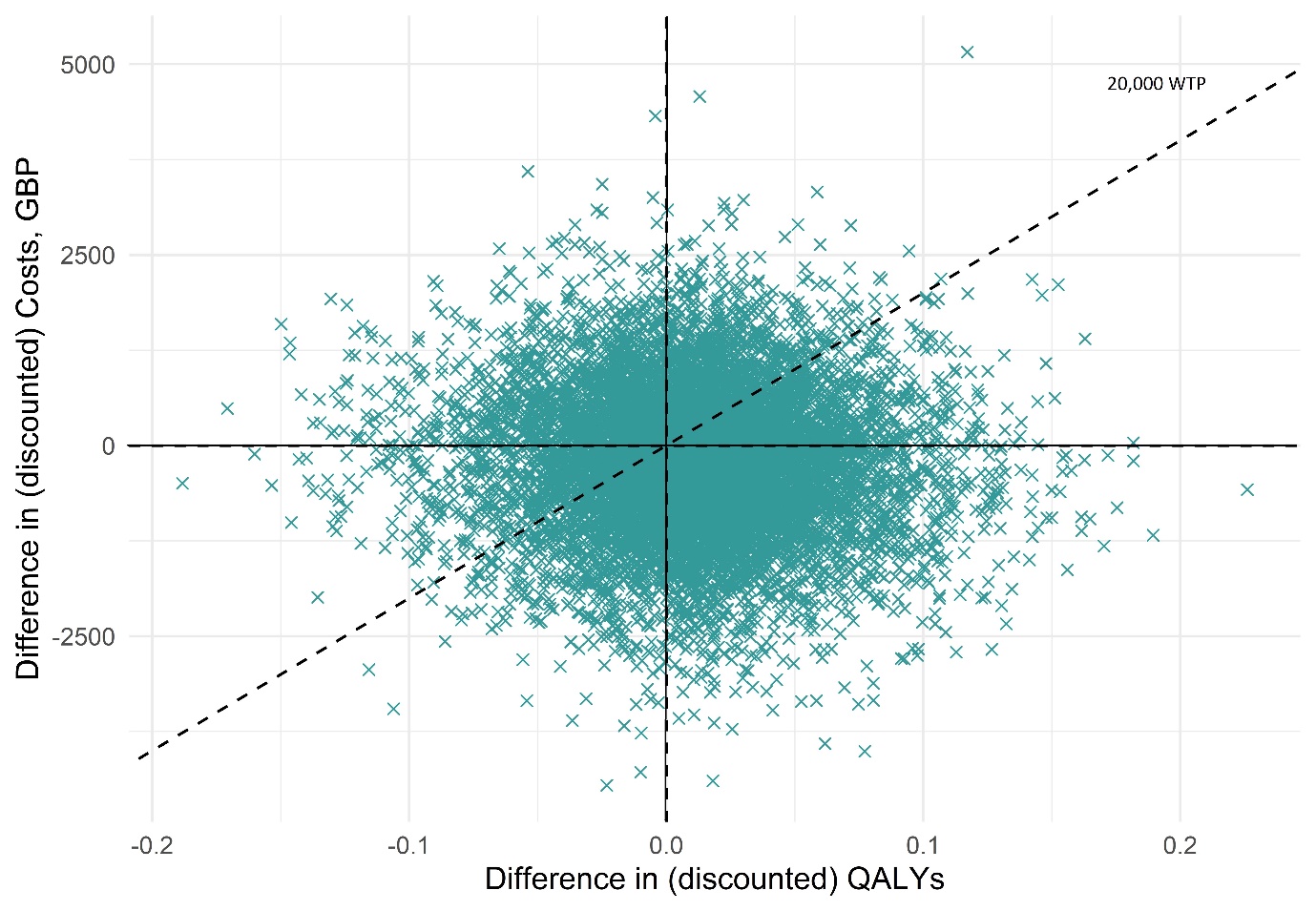


Scenario 1 - We tested the removal of the difference between the QALY estimates for the STAR care pathway and usual care for both CP and NCP. This was implemented to test the assumption made in the base case analysis that the health states (CP and NCP) QALY estimates would differ by comparator.

Figure A.3 Cost-effectiveness plane - scenario 2


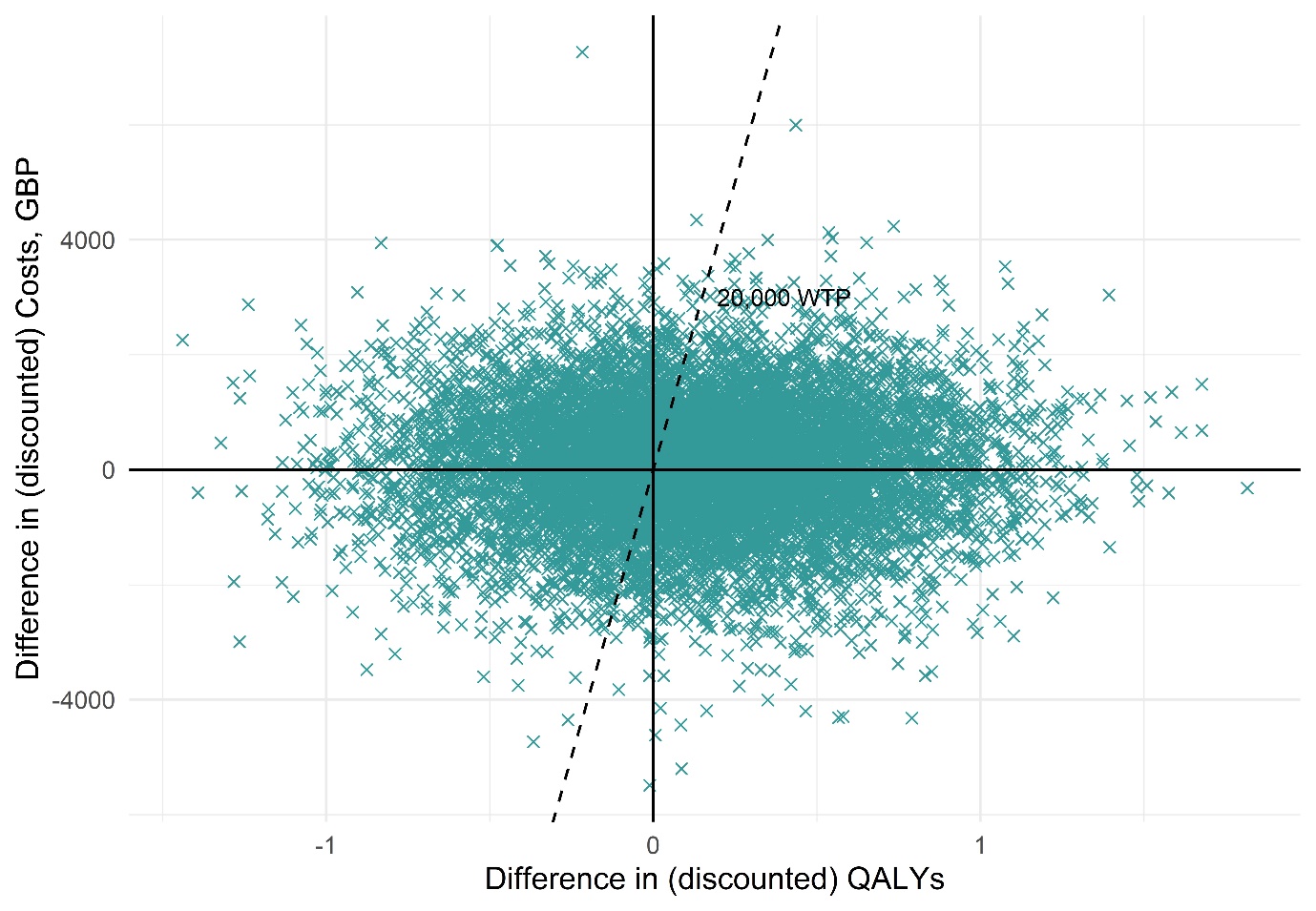


Scenario 2 - Considers that while in the trial, the intervention started at 12 weeks post-operation, the initial CP status was assessed at 10 weeks. This meant that two weeks later, at baseline, some patients could have moved out of CP. This scenario allows for some people to enter the model classified with NCP.

Figure A.4 Cost-effectiveness plane - scenario 3


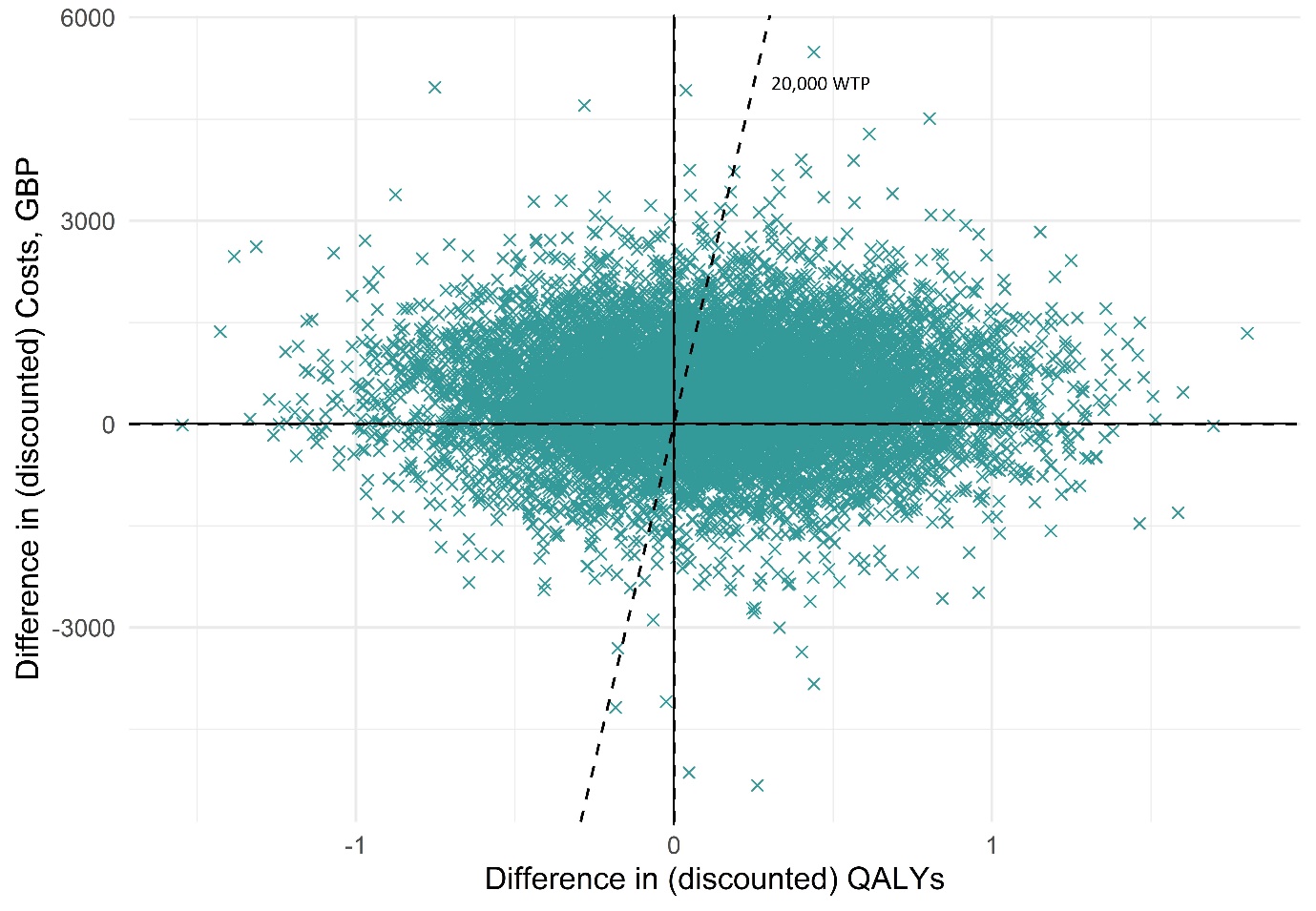


Scenario 3 - We considered hospital costs differing not by comparator but only by health states (CP and NCP) and for the first cycle. Hospital costs for all subsequent cycles (years 2-5) were estimated following the same approach as the base case.

**CHEERS 2022 Checklist**

| **Topic** | **No.** | **Item** | **Location where item is reported** |
| --- | --- | --- | --- |
| **Title** |  |  |  |
|  | 1 | Identify the study as an economic evaluation and specify the interventions being compared. | Title, page 1 |
| **Abstract** |  |  |  |
|  | 2 | Provide a structured summary that highlights context, key methods, results, and alternative analyses. | Abstract, pages 2-3 |
| **Introduction** |  |  |  |
| **Background and objectives** | 3 | Give the context for the study, the study question, and its practical relevance for decision making in policy or practice. | Pages 4-5 |
| **Methods** |  |  |  |
| **Health economic analysis plan** | 4 | Indicate whether a health economic analysis plan was developed and where available. | Methods, page 14 |
| **Study population** | 5 | Describe characteristics of the study population (such as age range, demographics, socioeconomic, or clinical characteristics). | Methods, page 6 |
| **Setting and location** | 6 | Provide relevant contextual information that may influence findings. | Methods, pages 6-7 |
| **Comparators** | 7 | Describe the interventions or strategies being compared and why chosen. | Methods, pages 5-8 |
| **Perspective** | 8 | State the perspective(s) adopted by the study and why chosen. | Methods, page 7 |
| **Time horizon** | 9 | State the time horizon for the study and why appropriate. | Methods, page 7 |
| **Discount rate** | 10 | Report the discount rate(s) and reason chosen. | Methods, page 7 |
| **Selection of outcomes** | 11 | Describe what outcomes were used as the measure(s) of benefit(s) and harm(s). | Methods, pages 9-11 |
| **Measurement of outcomes** | 12 | Describe how outcomes used to capture benefit(s) and harm(s) were measured. | Methods, pages 9-11 |
| **Valuation of outcomes** | 13 | Describe the population and methods used to measure and value outcomes. | Methods, pages 8-11 |
| **Measurement and valuation of resources and costs** | 14 | Describe how costs were valued. | Methods, page 10 |
| **Currency, price date, and conversion** | 15 | Report the dates of the estimated resource quantities and unit costs, plus the currency and year of conversion. | Methods, page 10 |
| **Rationale and description of model** | 16 | If modelling is used, describe in detail and why used. Report if the model is publicly available and where it can be accessed. | Methods, page 7/14 |
| **Analytics and assumptions** | 17 | Describe any methods for analysing or statistically transforming data, any extrapolation methods, and approaches for validating any model used. | Methods, pages 12-14 |
| **Characterising heterogeneity** | 18 | Describe any methods used for estimating how the results of the study vary for subgroups. | Not applicable |
| **Characterising distributional effects** | 19 | Describe how impacts are distributed across different individuals or adjustments made to reflect priority populations. | Methods, pages 12-13 |
| **Characterising uncertainty** | 20 | Describe methods to characterise any sources of uncertainty in the analysis. | Methods, pages 12-13 |
| **Approach to engagement with patients and others affected by the study** | 21 | Describe any approaches to engage patients or service recipients, the general public, communities, or stakeholders (such as clinicians or payers) in the design of the study. | Methods, page 8 |
| **Results** |  |  |  |
| **Study parameters** | 22 | Report all analytic inputs (such as values, ranges, references) including uncertainty or distributional assumptions. | Table 1 & Supplementary material |
| **Summary of main results** | 23 | Report the mean values for the main categories of costs and outcomes of interest and summarise them in the most appropriate overall measure. | Results, pages 14-15 |
| **Effect of uncertainty** | 24 | Describe how uncertainty about analytic judgments, inputs, or projections affect findings. Report the effect of choice of discount rate and time horizon, if applicable. | Results pages 15-16 |
| **Effect of engagement with patients and others affected by the study** | 25 | Report on any difference patient/service recipient, general public, community, or stakeholder involvement made to the approach or findings of the study | Not applicable |
| **Discussion** |  |  |  |
| **Study findings, limitations, generalisability, and current knowledge** | 26 | Report key findings, limitations, ethical or equity considerations not captured, and how these could affect patients, policy, or practice. | Discussion page 16-19 |
| **Other relevant information** |  |  |  |
| **Source of funding** | 27 | Describe how the study was funded and any role of the funder in the identification, design, conduct, and reporting of the analysis | Page 21 |
| **Conflicts of interest** | 28 | Report authors conflicts of interest according to journal or International Committee of Medical Journal Editors requirements. | Page 21 |

*From:* Husereau D, Drummond M, Augustovski F, et al. Consolidated Health Economic Evaluation Reporting Standards 2022 (CHEERS 2022) Explanation and Elaboration: A Report of the ISPOR CHEERS II Good Practices Task Force. Value Health 2022;25. <doi:10.1016/j.jval.2021.10.008>
